# Quantitative analytical technique applied to histopathology of birds infected experimentally by the virus of chicken anemia virus

**DOI:** 10.1186/1746-1596-3-S1-S21

**Published:** 2008-07-15

**Authors:** Luz García, Victor Bermudez, Mariela Brett, Luzmila Peroza, Juan Landa, Franklin Borregales

**Affiliations:** 1 Anatomic Pathology Lab, National Institute of Agricultural Research (INIA), AR, Venezuela; 2 Department of Pathology, Central University of Venezuela. School of Veterinary Medicine. AP-4563, AR, Venezuela

## Abstract

This research was conducted on ten glass slides selected from the histopathology evaluation chickens. Five slides of control's chickens healthy and five slides of chickens infected experimentally with chicken anemia virus (CAV slide) between one and twenty-one days post infection (PI), they were analyzed in magnifications of 200× and 400×. Histopathology showed severe bone marrow hypoplasia to complete aplasia, fully depletion of the erythrocytic and granulocytic series, both accompanied by space occupying adipocytic replacement. Foci of erythropoietic hyperplasia with intense mielopoietic activity, some hemocytoblast increased of size, with large nucleus. A quantitative analytical technique by Positive Pixel Count Algorithm was applied. It demonstrated that measures area stained of control slides were higher than CAV slides (Average: 61% vs. 25%, respectively). So, the control slides showed strong positivity, due to the presence of bigger quantity of cells of erythrocytic and granulocytic series. The CAV slides of seven days PI had high positivity (average: 94%), it was explained because the chicken anemia virus takes place severe lesions between ten to seventeen days PI, after 21 days PI the cellular regeneration is observed that is evidenced by means of focuses of erythroblastoid cells hyperplasia. This technique demonstrates in a quantitative way the severe decrease of the cellular components of the bone marrow in presence of the infection for CAV, supporting with numeric data the histology image evaluated by the pathologist. Therefore, it can be used as support to the histopathology of field samples to evaluate the presence of lesions taken place by CAV and this way to improve the quality and efficiency of the veterinary pathology services.

## Introduction

Chicken anemia virus (CAV) is a small, non-enveloped, single-stranded DNA virus, first isolated by Yuasa et al. (1979), a Gyrovirus, within the family Circoviridae [1]. The virus causes aplastic anaemia, generalized lymphoid atrophy and increased mortality after infection of day-old susceptible chickens [2]. The virus causes severe depletion of cortical thymocytes and erythroblastoid cells in the bone marrow, leading to immunodeficiency and anemia [3], because the VAIA lacks the necessary enzymes to reproduce its DNA in an independent way, it requires the cells to be divided very quickly [4]. The Positive Pixel Count is a multipurpose algorithm that measures area and intensity of staining for two-colored slides. This technique is useful as support to improve the histopathology evaluation making it objective by means of quantitative analytical techniques and this way, to improve the quality and efficiency of the pathology services. The purpose of this study was to apply quantitative analytical technique to histopathology of birds infected experimentally by the virus of chicken anemia virus.

## Materials and methods

### Samples

10 glass slides stained with H&E, selected of the histopathology evaluations of chickens. Five slides coming from chickens healthy controls and five slides coming from chickens infected experimentally with chicken anemia virus (CAV slide) between one and twenty one days PI, they were analyzed in m agnifications of 200× and 400×.

### Positive Pixel Count Algorithm (Aperio^®^)

Positive Pixel Count is a multipurpose algorithm that measures area and intensity of staining for two-colored slides. Pixels are classified according to colour and intensity of staining. Pixel counts (area) are calculated for strong positive (Red), positive (Orange), weak positive (Yellow), and negative (Blue) pixels. This is useful for measuring positivity as a fraction of total stained area.

## Results

**Figure 1:**
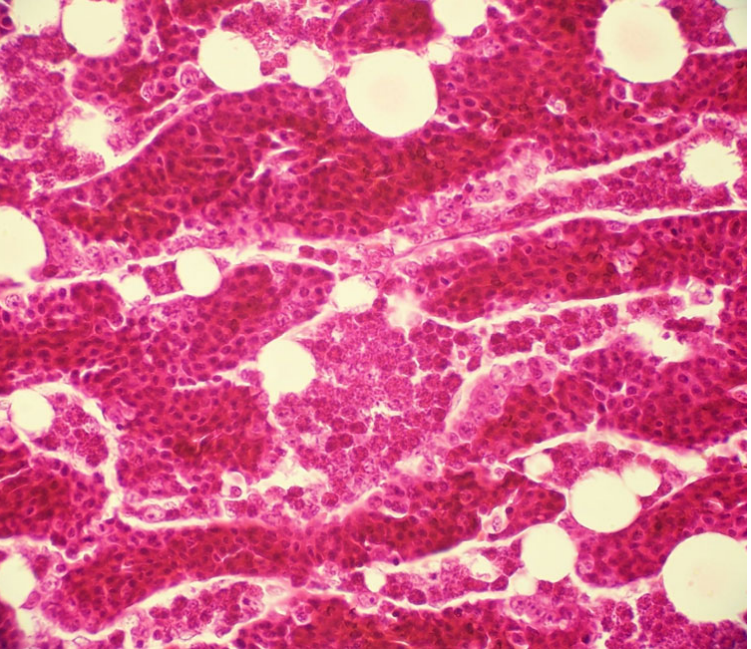
Bone marrow of control bird 10 days age (400×, H&E).

**Figure 2:**
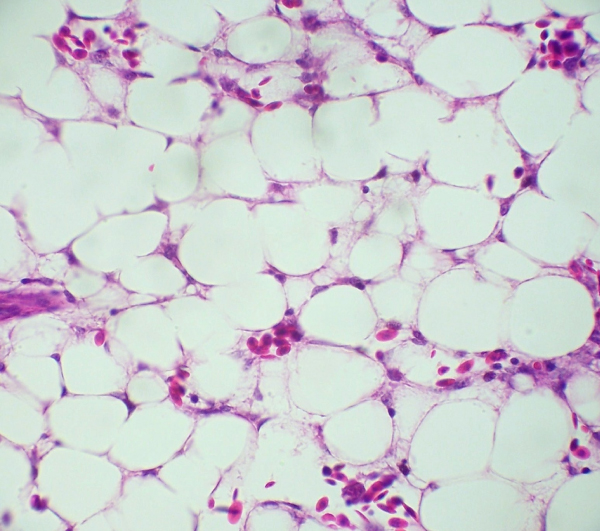
Bone marrow of bird infected with CAV 10 days PI (400×, H&E).

Histopathology showed bone marrow with abundant cells of the erythrocytic and granulocytic series from chickens healthy controls (Figure 1) and severe bone marrow hypoplasia and/or complete aplasia, fully depletion of the erythrocytic and granulocytic series, both accompanied by space occupying adipocytic replacement (Figure 2) in the birds of 10, 14, 17 and 21 days after inoculation. A quantitative analytical technique by Positive Pixel Count Algorithm was applied. The data are shown below in Table 1.

**Table 1:** Number of Strong Positive and Positivity (%) of Control slides and CAV slides in magnification 200× and 400×.

		CONTROL		CAV	
		
DAYS PI	Magnification ×	Number of Strong Positive	Positivity (%)	Number of Strong Positive	Positivity (%)
7	200	343704	86%	20215	91%
	400	507440	96%	184450	97%
10	200	380520	54%	4112	18%
	400	317731	100%	675	11%
14	200	264432	41%	2505	5%
	400	506791	28%	5156	9%
17	200	138707	40%	46	4%
	400	147699	89%	6262	2%
21	200	274310	29%	13926	10%
	400	589143	43%	217787	8%

Average		320148,22	61%	26371,88	25%

## Discussion

The results demonstrated that the number of Strong Positive and Positivity (%) of control slides were higher than CAV slides (Average: 61% vs. 25%, respectively). The control slides showed strong positivity, due to the presence of bigger quantity of erythroblastoid cells. The CAV slides of 7 days PI had high positivity (average: 94%), because the CAV produces the lesions but severe starting from the 10 days PI up 21 days PI, after the cellular regeneration is observed, being able to be evidenced by means of focuses of erythroblastoid cells hyperplasia.

## Conclusion

This technique demonstrated in a quantitative way the severe decrease of the cellular components of the bone marrow in presence of the infection for CAV, supporting with numeric data the histology image evaluated by the pathologist. Therefore, it can be used as support to the histopathology of field samples to evaluate the presence of lesions taken place by CAV in the farms and this way to improve the quality and efficiency of the veterinary pathology services.

## References

[B1] Pringle CR (1999). Virus taxonomy at the XIth International Congress of Virology. Arch Virol.

[B2] Engstrom BE, Luthman M (1984). Blue wing disease of chickens: experimental infection with a Swedish isolate of chicken anaemia agent and an avian reovirus. Avian Pathol.

[B3] Noteborn MHM, Koch G (1995). Chicken anaemia virus infection: molecular basis of pathogenicity. Avian Pathol.

[B4] Schat K (2005). Traducción: Dr. Néstor A. Mossos. Instituto Colombiano Agropecuario – ICA. El virus de La Anemia Infecciosa Aviar (VAIA): un patógeno inmunosupresor de gran importancia.

[B5] Yuasa N, Tanigushi T, Yoshida I (1979). Isolation and some characteristics of and agent inducing anemia in chicks. Avian Dis.

